# Successful extraction of a large airway foreign body using flexible bronchoscopy and electrocautery snare in a post-COVID-19 patient with difficult airway anatomy: a case report

**DOI:** 10.3389/fmed.2025.1600828

**Published:** 2025-06-09

**Authors:** Yugang Lu, Jiawei Dong, Ye Gu

**Affiliations:** ^1^Department of Anesthesiology, Shanghai Pulmonary Hospital, Tongji University School of Medicine, Shanghai, China; ^2^Department of Endoscopy Center, Shanghai Pulmonary Hospital, Tongji University School of Medicine, Shanghai, China

**Keywords:** airway foreign bodies, rigid bronchoscopy, flexible bronchoscopy, short thyromental distance, electrocautery snare, general anesthesia (GA)

## Abstract

A 64-year-old man presented with a 10-cm metal spoon handle retained in his airway for 40 years—a rare case of chronic foreign body aspiration in an adult. The condition was further complicated by post-COVID-19 respiratory symptoms and challenging airway anatomy, including a short thyromental distance and Mallampati Class IV classification. The patient complained of persistent chest tightness, shortness of breath, and recurrent respiratory issues that persisted after recovering from COVID-19. Initial attempts at removal using rigid bronchoscopy (RB) failed due to anatomical limitations. However, the foreign body was successfully extracted via flexible bronchoscopy (FB) using an electrocautery snare, without airway injury or bleeding. The patient's symptoms resolved immediately, and he was discharged within 24 h, showing sustained improvement at a 3-month follow-up. This case underscores the importance of pre-procedural airway assessment to anticipate technical challenges and the need for procedural adaptability. When RB fails, FB with advanced tools such as electrocautery snares can serve as an effective alternative. RB and FB should be seen as complementary techniques, and clinical teams should be prepared to use both, along with appropriate innovations. Moreover, the case highlights FB's expanding role in managing complex, chronic airway foreign bodies and the critical role of flexibility, planning, and specialized tools in achieving optimal outcomes.

## Introduction

Airway foreign body aspiration in adults is relatively uncommon compared to children, with an estimated annual incidence of 0.2–1.5 per 100,000 adults. Risk factors include impaired swallowing (e.g., neurological disorders and alcohol/drug use), dental procedures, or eating habits (e.g., hurried eating) (1, 2). Airway foreign bodies usually cause chronic cough, recurrent pneumonia, and atelectasis, and in severe cases, they can be fatal (3). Bronchoscopy, a procedure that allows for visualization and extraction of foreign bodies from the airway, is the standard approach for managing these cases. It can be performed using either rigid or flexible bronchoscopy. Flexible bronchoscopy (FB) is suitable for small, flat, or round objects, while rigid bronchoscopy (RB) is preferred for larger, sharp-edged, or long-retained foreign bodies (4). Due to its dual role in airway management and foreign body removal, providing powerful control and the ability to use large instruments (5), RB is the traditional method for extracting complex airway foreign bodies. However, a significant proportion of cases may require conversion from FB to RB (5). Conversely, when conversion from RB to FB is necessary, the procedure faces greater challenges and increased risks.

In this study, we report a rare case of successful removal of a large airway foreign body, retained for 40 years, using an electrocautery snare via FB after the failure of RB insertion. Written informed consent was obtained from the patient, and approval was granted by the Research Ethics Committee of our hospital for medical education and publication.

## Case description

**Figure 1:**
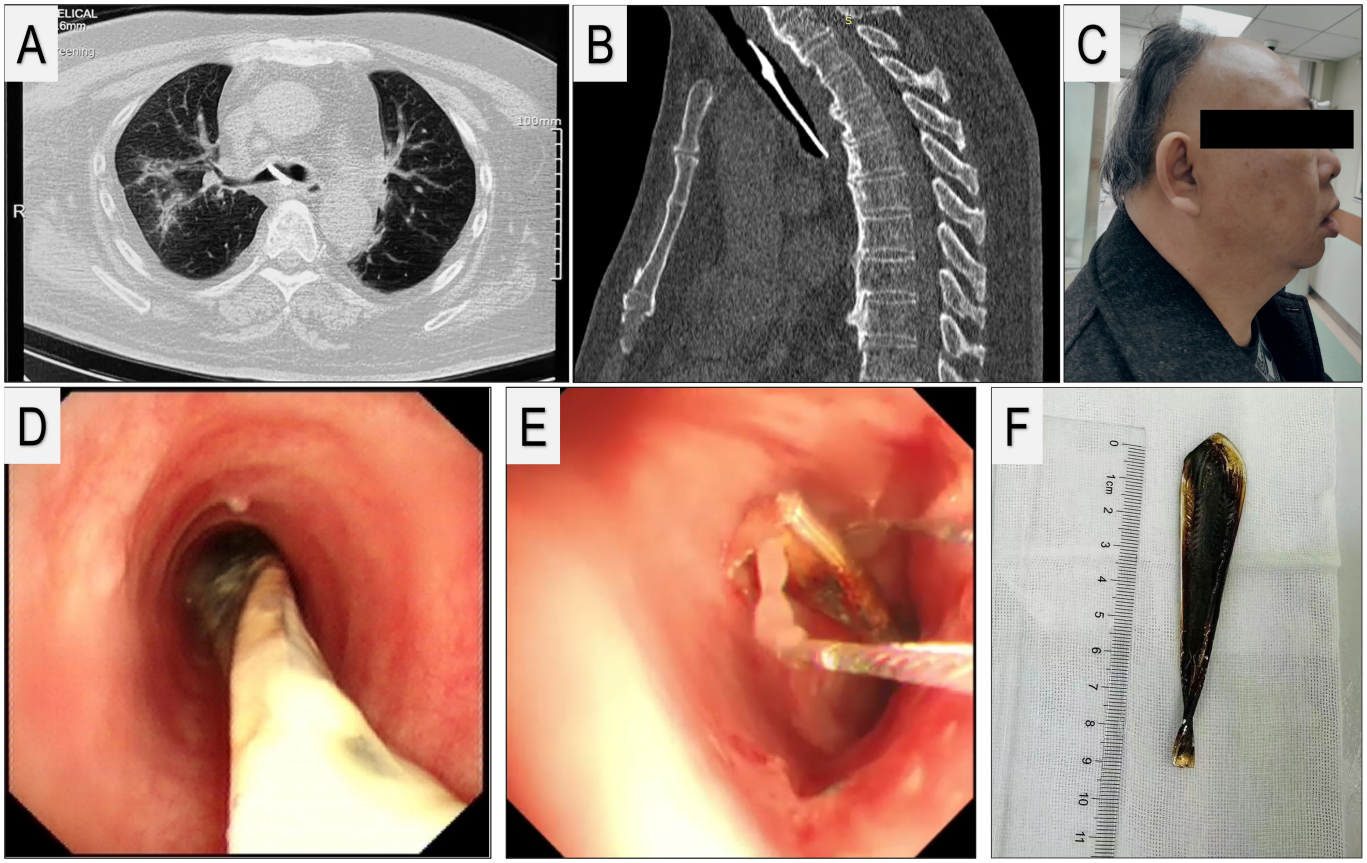
**(A)** CT scan of the chest showing the lower end of the foreign body was inserted into the right main bronchus and bronchopneumonia lesion. **(B)** A sagittal plane of the CT scan of the chest showing the location of the foreign body in the trachea. **(C)** Right-side view of the patient's facial appearance, accompanied by the Mallampati score IV and thyromental distance: 3 fingerbreadths. **(D)** Initial bronchoscopy view of the foreign body occluding the trachea. **(E)** Ligation of the upper segment of the foreign body with a snare via flexible bronchoscopy. **(F)** The foreign body, a metal spoon handle, ~10 cm long.

A 64-year-old male patient presented with persistent chest tightness and shortness of breath after full recovery from severe COVID-19. The patient, with a BMI of 30 kg/m^2^, was initially diagnosed with COVID-19 in December 2020 and was rapidly transferred to the ICU due to pneumonia and respiratory failure. The chest high-resolution computed tomography during hospitalization is shown in Figure 1A. The patient received standard antiviral and respiratory support therapy for 2 weeks and achieved a full recovery, as confirmed by imaging and testing results. However, the respiratory system symptoms persisted or even aggravated for months after discharge, which was inconsistent with his testing results. The patient recalled an incident of accidental foreign body aspiration 40 years ago. Previous CT scans also revealed an airway foreign body located at the lower right main bronchial orifice (Figures 1A, B), which was considered the likely cause of his ongoing symptoms. Consequently, we scheduled an airway foreign body extraction using rigid bronchoscopy (RB) under general anesthesia.

Upon admission, the patient was in a stable condition, and there was no decrease in oxygen saturation. Physical examination revealed a thyromental distance (TMD) <6.5 cm (Figure 1C) with Mallampati Class IV, indicating a potentially difficult airway and hardness for RB. Standard monitoring, including electrocardiogram, pulse oximetry, and non-invasive blood pressure, was applied. A peripheral intravenous catheter was placed, and Ringer's solution infusion was started in the operating room. Anesthesia induction was performed using standard doses of remimazolam, propofol, alfentanil, and rocuronium bromide, with maintenance achieved through total intravenous anesthesia.

After general anesthesia, the endoscopists tried multiple times to insert the RB but failed. Consequently, the procedure was converted from the RB to the FB. Under bronchoscopy, the upper end of the foreign body was observed in the upper segment of the trachea, and the lower end was inserted into the right main bronchus, with partial airway obstruction (Figure 1D). Initially, endoscopic foreign body retrieval forceps were used; however, the foreign body was a long metal strip, which was difficult to take out through the glottis without hurting the bronchus or surrounding tissue. In addition, adjusting the angle while keeping the foreign body beneath the glottis quickly resulted in hypoventilation and hypoxemia. Thus, the operator had to reinsert the strip into the bronchus.

Ultimately, an electrocautery snare was chosen due to its ability to securely grasp the upper end of the foreign body and prevent dislodgement during angle adjustment. The snare was tethered to the upper part of the foreign body, which was then softly extracted from the trachea into the laryngeal mask (Figure 1E), and the foreign body, along with the laryngeal mask, was removed together (Supplementary Video 1). Afterward, the laryngeal mask was reinserted, and bronchoscopy was performed for a checkup. No glottic injury, airway mucosal damage, or bleeding was observed. After recovery of consciousness and removal of the laryngeal mask, the patient was transferred to the post-anesthesia care unit.

The foreign body, a metal spoon handle (Figure 1F) with a length of nearly 10 cm, had been stuck in the patient's airway for more than 40 years and was successfully removed. The patient's symptoms were relieved after the procedure, and he was discharged on the first postoperative day. Clinical improvements were well-maintained for a follow-up of 3 months. The patient expressed sincere gratitude for the treatment provided.

## Discussion

We present a rare case of a 64-year-old man with a 10-cm metal spoon handle lodged in his airway. The patient, who had a short thyromental distance, underwent successful extraction of the foreign body using an electrocautery snare via FB, with no complications occurring during or after the procedure.

Although RB is generally considered the standard approach for removing large, sharp-edged, or long-standing intrabronchial foreign bodies, the patient's anatomical limitations (short thyromental distance) and the unsuccessful attempts to insert the RB prompted a switch to FB. With FB, we were able to assess the general morphology and location of the foreign body. An initial attempt at removal using endoscopic foreign body retrieval forceps was unsuccessful due to the forceps' inability to securely grasp the foreign body, which subsequently became lodged at the glottis, obstructing ventilation. This led to a progressive decline in the patient's oxygen saturation levels. Ultimately, we successfully extracted the foreign body using the electrocautery snare via FB. This case underscores the importance of a thorough preoperative airway assessment, the availability of multiple solutions, and the flexibility to switch between tools as needed. It emphasizes the complementary, rather than competitive, relationship between FB and RB in managing complex airway foreign body cases.

The thyromental distance (TMD), defined as the distance from the mentum (chin) to the top of the notch of the thyroid cartilage with the neck in full extension, is a critical anatomical parameter in airway management. It plays a significant role in predicting difficult intubation and airway interventions, including RB. TMD reflects the space available for aligning the oral, pharyngeal, and laryngeal axes during airway manipulation. A short TMD of <6.5 cm has been shown as a predictive factor in difficult intubation (6, 7), especially if the Mallampati score is III or IV (8). RB demands optimal neck extension to align the oral, pharyngeal, and tracheal axes. A short TMD limits cervical spine mobility, making it challenging to achieve the “sniffing position” required for RB. This may lead to prolonged procedure time or insertion failure (9). In addition, in patients with a small TMD, forceful manipulation during RB insertion may increase the risk of dental injury, laryngeal trauma, or bleeding, particularly if rescue devices (e.g., McCoy laryngoscope) are not utilized. Therefore, TMD should be routinely measured alongside other predictors (e.g., Mallampati score, cervical mobility, and BMI) in patients scheduled for RB. A short TMD is a “key red flag” for difficult RB, necessitating tailored airway strategies. While not definitive alone, its integration into comprehensive airway assessment significantly enhances procedural safety and success rates.

The management of tracheobronchial foreign bodies (TFBs) often requires prompt intervention to prevent life-threatening complications such as airway obstruction, pneumonia, or perforation. While rigid bronchoscopy remains the gold standard for large or complex foreign body removal due to its superior airway control and versatility with instrumentation, failures may occur due to anatomical challenges, operator inexperience, or the location/composition of the foreign body. In such scenarios, FB emerges as a viable alternative. With the advancement of techniques and instruments, successful TFB retrieval by FB has gradually increased.

Fogarty catheters, ureteral stone baskets, biopsy forceps, and alligator forceps have been reported to be used for the retrieval of TFBs (4, 10, 11). Due to weaker tips, Eedoh et al. used 3-pronged foreign body grasping forceps instead of standard grasping forceps for TFB retrieval via FB (12). In addition, cryoprobes and endobronchial blockers have also been reported to be used for the removal of TFBs via FB (13, 14).

The electrocautery snare, a tool traditionally used for endoscopic resection of polyps or tumors, has emerged as a valuable adjunct in TFB extraction, particularly for challenging cases where conventional forceps or baskets fail (15, 16). Its ability to combine mechanical capture with thermal energy allows for the controlled removal of impacted or embedded objects while minimizing bleeding. Guidelines from the American Association for Bronchology and Interventional Pulmonology (AABIP) highlight the electrocautery snare as a tier-2 option for complex TFBs, emphasizing operator experience. Wire loop and polypectomy snares have been reported to be used for the successful extraction of TFBs via FB (16, 17). In our case, after an unsuccessful attempt to remove the metal spoon handle with foreign body forceps, we decisively opted for the electrocautery snare, which securely grasped one end of the foreign body and successfully maneuvered it past the glottis, ultimately leading to its successful extraction.

This case is notable for the rare presentation of a 10-cm metal foreign body retained for 40 years and its innovative management using FB with an electrocautery snare, overcoming anatomical constraints that precluded RB. The successful extraction demonstrates procedural adaptability, the repurposing of tools, and the complementary roles of RB and FB. However, as a single-case report, it has limited generalizability, and the 3-month follow-up is insufficient to assess long-term outcomes, such as airway remodeling or recurrence. Broader conclusions will require validation through larger, multicenter studies.

Key clinical takeaways include the following:

1. Preoperative airway assessment is crucial: measure TMD and Mallampati score to predict difficult airway interventions. A short TMD (<6.5 cm) with Mallampati Class III/IV indicates a high risk for RB failure.
2. FB as a rescue tool: when RB is not feasible, FB with advanced instruments (e.g., electrocautery snare) can safely manage large, chronic foreign bodies. Familiarity with adjunct tools (e.g., snares and cryoprobes) enhances FB's utility.
3. Creative tool repurposing: electrocautery snares, typically used for polypectomy, can securely grasp rigid or slippery foreign bodies while minimizing mucosal trauma.

In managing complex airway foreign bodies, integrate preoperative airway evaluation with the flexibility to switch between RB and FB. Equip your team with versatile tools (e.g., electrocautery snares) and emphasize adaptive strategies—complementing RB's strengths with FB's flexibility to optimize outcomes in anatomically challenging cases. This case encourages clinicians to view RB and FB as complementary, rather than competing, approaches and to develop expertise in advanced FB techniques.

This report provides insights into the extraction of a 10-cm metal spoon handle using an electrocautery snare via FB in a patient with a short TMD, offering guidance to pulmonologists, anesthesiologists, and endoscopists in managing endobronchial foreign body removal.

## Data Availability

The raw data supporting the conclusions of this article will be made available by the authors, without undue reservation.

## Supplementary material

Video 1 Extraction of the large airway foreign body with an electrocautery snare via flexible bronchoscopy under general anesthesia.

## References

[1] Zhan J Du Y Wu J Lai F Song R Wang Y. The global, regional, and national burden of foreign bodies from 1990 to 2019: a systematic analysis of the global burden of disease study 2019. BMC Public Health. (2024) 24:337. 10.1186/s12889-024-17838-x 38297245 PMC10829478

[2] Sehgal IS Dhooria S Ram B Singh N Aggarwal AN Gupta D. Foreign body inhalation in the adult population: experience of 25,998 bronchoscopies and systematic review of the literature. Respir Care. (2015) 60:1438–48. 10.4187/respcare.03976 25969517

[3] Wang Y Wang J Pei Y Qiu X Wang T Xu M. Extraction of airway foreign bodies with bronchoscopy under general anesthesia in adults: an analysis of 38 cases. J Thorac Dis. (2020) 12:6023–9. 10.21037/jtd-20-2903 33209435 PMC7656387

[4] Suzen A Karakus SC Erturk N. The role of flexible bronchoscopy accomplished through a laryngeal mask airway in the treatment of tracheobronchial foreign bodies in children. Int J Pediatr Otorhinolaryngol. (2019) 117:194–7. 10.1016/j.ijporl.2018.12.006 30579081

[5] Safia A Abd Elhadi U Bader R Khater A Karam M Bishara T. Flexible vs. rigid bronchoscopy for tracheobronchial foreign body removal in children: a comparative systematic review and meta-analysis. J Clin Med. (2024) 13:5652. 10.3390/jcm13185652 39337140 PMC11433179

[6] Qudaisat IY Al-Ghanem SM. Short thyromental distance is a surrogate for inadequate head extension, rather than small submandibular space, when indicating possible difficult direct laryngoscopy. Eur J Anaesthesiol. (2011) 28:600–6. 10.1097/EJA.0b013e328347cdd9 21610502

[7] Sharma V Yadav HP Prakash A Yadav N Kumar M Abbas H. Assessment of different indices as predictors of difficult airway in obese patients. Cureus. (2024) 16:e55005. 10.7759/cureus.55005 38414514 PMC10897764

[8] Patel B Khandekar R Diwan R Shah A. Validation of modified Mallampati test with addition of thyromental distance and sternomental distance to predict difficult endotracheal intubation in adults. Indian J Anaesth. (2014) 58:171–5. 10.4103/0019-5049.130821 24963182 PMC4050934

[9] Ads A Auerbach F Ryan K El-Ganzouri AR. Air-Q laryngeal airway for rescue and tracheal intubation. J Clin Anesth. (2016) 32:108–11. 10.1016/j.jclinane.2016.02.004 27290957

[10] Martinez-Pozas O Corbellini C Cuenca-Zaldivar JN Melendez-Oliva E Sinatti P Sanchez Romero EA. Effectiveness of telerehabilitation vs. face-to-face pulmonary rehabilitation on physical function and quality of life in people with post COVID-19 condition: a systematic review and network meta-analysis. Eur J Phys Rehabil Med. (2024) 60:868–77. 10.23736/S1973-9087.24.08540-X 39235257 PMC11561472

[11] Sanchez Romero EA Alonso Perez JL Vinuesa Suarez I Corbellini C Villafane JH. Spanish experience on the efficacy of airways clearance techniques in SARS-CoV-2 (COVID-19) at intensive care unit: an editorial and case report. SAGE Open Med Case Rep. (2022) 10:2050313X221112507. 10.1177/2050313X221112507 35875169 PMC9297451

[12] Endoh M Oizumi H Kanauchi N Kato H Ota H Suzuki J. Removal of foreign bodies from the respiratory tract of young children: treatment outcomes using newly developed foreign-body grasping forceps. J Pediatr Surg. (2016) 51:1375–9. 10.1016/j.jpedsurg.2016.02.045 27001457

[13] Sehgal IS Dhooria S Behera D Agarwal R. Use of cryoprobe for removal of a large tracheobronchial foreign body during flexible bronchoscopy. Lung India. (2016) 33:543–5. 10.4103/0970-2113.188978 27625452 PMC5006338

[14] Litman RS Ponnuri J Trogan I. Anesthesia for tracheal or bronchial foreign body removal in children: an analysis of ninety-four cases. Anesth Analg. (2000) 91:1389–91. 10.1097/00000539-200012000-00015 11093985

[15] Weber SM Chesnutt MS Barton R Cohen JI. Extraction of dental crowns from the airway: a multidisciplinary approach. Laryngoscope. (2005) 115:687–9. 10.1097/01.mlg.0000161353.45999.dd 15805882

[16] Tu CY Chen HJ Chen W Liu YH Chen CH A. feasible approach for extraction of dental prostheses from the airway by flexible bronchoscopy in concert with wire loop snares. Laryngoscope. (2007) 117:1280–2. 10.1097/MLG.0b013e3180581991 17603327

[17] Tenenbaum T Kahler G Janke C Schroten H Demirakca S. Management of foreign body removal in children by flexible bronchoscopy. J Bronchology Interv Pulmonol. (2017) 24:21–8. 10.1097/LBR.0000000000000319 27623415

